# The Challenges and Therapeutic Prospects in Eye Disease

**DOI:** 10.3390/jpm13060930

**Published:** 2023-05-31

**Authors:** Chieh-Chih Tsai

**Affiliations:** 1 Department of Ophthalmology, Taipei Veterans General Hospital, Taipei 11217, Taiwan; cctsai@vghtpe.gov.tw; 2 School of Medicine, National Yang Ming Chiao Tung University, Taipei 11221, Taiwan

A number of key insights into eye disease have been revealed in the past decade, which has resulted in the development of novel, effective, targeted therapies such as teprotumumab for the treatment of thyroid eye disease (also known as Graves’ orbitopathy) [1] and intraocular injections of antivascular endothelial growth factor (VEGF) agents for many retinal diseases [2]. This Special Issue aims to update the current evidence regarding challenging topics on the diagnosis and management of ocular diseases. We collected ten original research articles and two literature reviews on recent efforts made toward the discovery of novel findings in different ocular research areas, including orbital, lacrimal, and eyelid disease; glaucoma; retinal disease; myopia; and corneal disease.

**Orbital, lacrimal, and eyelid disease**

IgG4-related diseases (IgG4-RDs) are an emerging fibro-inflammatory condition that can be characterized by affected organ enlargement, infiltration of lymphocytes and IgG4-positive plasma cells in various tissues and organs, and serum IgG4 level elevation [3]. Serial changes in serum IgG4 levels is a useful adjunct diagnostic method in the assessment of disease activity in patients with IgG4-ROD [4]. Chou et al. further investigated the relationship between the long-term changes in serum IgG4 levels and the clinical course of patients with IgG4-ROD [5]. They found 64% patients with IgG4-ROD had persistently high serum IgG4 levels during long-term follow-up. Importantly, their study disclosed 40% of patients with IgG4-ROD remained in remission despite persistently elevated serum IgG4 levels. Chou et al. suggest these patients can be followed-up without treatment unless disease relapse occurs [5].

Hydrogel scleral buckles were first introduced in the 1980s as an alternative to silicone buckles for the treatment of rhegmatogenous retinal detachment. Hydrogel scleral buckles may absorb tissue fluids and progressively expand over the decades, causing compression of the eyeball, and are commonly misdiagnosed as orbital tumors [6]. The removal of swelling hydrogel scleral buckles is challenging due to their fragile characteristics and fibrotic adhesion to the surrounding ocular tissues. Yang et al. introduced a small excision surgical technique to meticulously remove swelling hydrogel scleral buckles to avoid the risk of severe complications associated with manipulations [7].

Graves’ orbitopathy (GO), an increase in orbital fat and/or muscle tissue due to autoimmune inflammation, can cause proptosis, double vision, and/or dysthyroid optic neuropathy (DON). Steiert et al. examined visual parameters dependent on the orbital muscle volume fraction in a surgically treated GO cohort and suggested that the orbital muscle volume factor should be addressed during treatment decisions, while early orbital decompression should be considered particularly in decreased vision with orbital muscle enlargement [8]. In a way that is different to the traditional staged GO management order (decompression and then muscle surgery or lid surgery), Hsieh et al. suggested that orbital decompression combined with strabismus surgery by experienced surgeons can achieve satisfactory outcomes in selected patients, especially for those with symmetry of orbitopathy, relatively simple strabismus and mild proptosis [9]. Choi et al. further supported the use of customized orbital decompression surgery combined with eyelid or strabismus surgery in mild to moderate GO patients, as it had favorable cosmetic results comparable to those achieved through stepwise techniques [10].

Balloon dacryocystoplasty is a minimally invasive procedure that can achieve lacrimal system patency and is usually performed mainly in cases of congenital nasolacrimal duct obstruction with failed probing [11]. It is known to be more effective than probing alone in older children after 18 months of age with congenital nasolacrimal duct obstruction [12]. It may also be used for the treatment of adult patients with partial nasolacrimal duct obstruction, especially in those who are poor candidates for dacryocystorhinostomy or general anesthesia [13]. Lai et al. found that balloon dacryocystoplasty (DCP) with pushed monocanalicular intubation in adults aged under 65 with complete primary acquired nasolacrimal duct obstruction can achieve a higher success rate than those who underwent balloon dacryocystoplasty alone [14].

Blepharoptosis, the drooping of the upper eyelid, may cause upper visual defects and affect vision. It is important for general practitioners (nonophthalmic physicians) to correctly diagnosis blepharoptosis to assist in decision making for referrals and/or advance work-ups when necessary. Medical artificial intelligence (AI) using machine learning and deep learning has been adopted by various groups to provide effective solutions to challenges facing ophthalmologists and healthcare providers worldwide. Hung et al. developed an AI model with a convolutional neural network (CNN)-based deep learning method to assist in the diagnosis of blepharoptosis, and they demonstrated that the AI model showed better performance than the nonophthalmic physician group in identifying referable blepharoptosis, including true ptosis and pseudoptosis [15].

**Glaucoma**

Glaucoma is a neurodegenerative disease that affects primarily the retinal ganglion cells (RGCs). Reductions in IOP, achieved with medication, laser, or surgery, has until now been considered as the only effective treatment strategy for slowing down or halting glaucoma progression. However, an even lower IOP does not preclude the possibility of glaucoma progression, as showed by the Collaborative Normal-Tension Glaucoma Study [16]. More and more ophthalmic researchers have tried to develop neuroprotective therapies as a supplement to IOP-lowering treatment. Kuo et al. summarized recent therapeutic advances in IOP-independent neuroprotection research, and discussed the feasibility and hurdles of each potential therapeutic mechanism of various agents in neuroprotection [17]. Nocturnal intraocular pressure (IOP) fluctuations are an important issue in the management of glaucoma. Huang et al. attempted to use a contact lens sensor to monitor the IOP circadian fluctuation change after ripasudil eye drop administration [18]. Although the value of each parameter became lower after the use of ripasudil eye drops, the reduction did not reach statistical significance due to the low baseline IOP in patients with normal tension glaucoma.

**Retina**

Age-related macular degeneration (AMD), a degenerative disease of the macula, is the most prevalent retinal disease in the Western world, and the neovascular form of AMD may lead to progressive vision loss and cause impairment of the quality of life [19]. Advances in multimodal imaging techniques, fluorescein angiography, indocyanine angiography, optical coherence tomography (OCT), and OCT angiography have been developed for the early diagnosis and follow-up of patients with AMD. Kirkova et al. used OCT-A to provide prognostic markers and new treatment strategies for AMD depending on the naïve neovascular membrane morphologic type [20]. Chen et al. further stated that OCT morphological changes in patients with diabetic macular edema receiving an intravitreal injection of ranibizumab was correlated with a worse structural and visual outcome; thus, serous retinal detachment may be an indicator of an earlier stage of diabetic macular edema, which responds well to the intravitreal injection of ranibizumab [21].

**Refractive errors**

Increasing studies have revealed that changes in the bioelectrical activity of masticatory and cervical spine muscles may have a primary or secondary effect on the refractive error. [22,23,24]. Zieliński et al. further showed refractive errors are related to differences in masticatory and neck muscle thickness, and the activity and bioelectrical activity within the temporalis anterior seems to be associated with ocular length, retinal thickness, and choroidal thickness in women with myopia [25]. This may support the finding of interdependence between the stomatognathic system and the organ of vision.

**Cornea**

Photoactivated chromophore corneal collagen cross-linking (PACK-CXL) is widely known as a minimally invasive therapy for corneal ectasia via stiffening of the cornea tissue by combining ultraviolet A radiation and riboflavin (vitamin B2) through collagen fiber photopolymerization. [26,27] PACK-CXL has also been proposed to be an emerging application to treat infectious keratitis. Infectious keratitis is associated with a risk of visual loss and is one of the leading causes of blindness in developing countries. Infectious keratitis has been conventionally treated with topical broad-spectrum antibiotics or antifungals. PACK-CXL offers a potential, less expensive alternative, acting as a primary or adjunctive treatment for infectious keratitis. Barac et al. carried out a comprehensive review to identify the main diagnostic and prognostic factors involved in therapeutic indications and contraindications of PACK-CXL in infectious keratitis [28].

In conclusion, all articles appearing in this Special Issue provide attractive and current topics that cover a wide range of clinical diagnosis, prognosis, and therapeutic strategies for various ocular diseases. In the future, more investigations will continue to drive our progress toward improving the diagnosis and treatments to meet the challenges and future prospects of eye disease.
